# Intensive management negatively impacts field margin ecosystem service indicators at both field and landscape levels

**DOI:** 10.1002/eap.70161

**Published:** 2026-01-06

**Authors:** Léa Genty, Christine N. Meynard, Marie‐Charlotte Bopp, Laura Henckel, Aurélien Chayre, Caroline Gibert, Guillaume Fried

**Affiliations:** ^1^ Anses Laboratoire de la Santé des Végétaux, Unité Entomologie et Botanique Montferrier‐sur‐Lez France; ^2^ CBGP INRAE, CIRAD, Institut Agro, IRD, University of Montpellier Montpellier France; ^3^ Agroécologie INRAE, Institut Agro, University of Bourgogne, University of Bourgogne Franche‐Comté Dijon France; ^4^ Agence Régionale de la Biodiversité Bourgogne‐Franche‐Comté Besançon France; ^5^ Solagro Agroécologie Biodiversité Toulouse France

**Keywords:** ecosystem services, erosion, field margin vegetation, floral resources, pesticides, plant conservation, weeds

## Abstract

Vegetated field margins generally increase plant biodiversity and connectivity in agricultural landscapes. They can deliver ecosystem services, such as providing food and shelter for insects, or maintaining biotic regulation. But they can also represent a risk, for example by hosting competitor plants or cultivated crop pests. In this work, we evaluated the effects of agricultural practices on indicators of three ecosystem services (providing floral resources for pollinators, reducing soil erosion and conserving plant biodiversity), and one ecosystem disservice (competing with the crop by hosting problematic weeds). We used a French nationwide‐scale monitoring network, composed of more than 450 fields of cereals, vineyards, and market gardening. Plant sampling and agricultural practices surveys were conducted from 2013 to 2018. We unambiguously found that pesticide use, at either field or municipality levels, or both, had detrimental effects on ecosystem service indicators. Herbicide use and fertilization quantity decreased floral resources, affecting both their quantity and diversity. Pesticide use was also associated with fewer nature‐value species and more problematic weeds. Margin management could also sometimes affect the service and disservice indicators. This work not only increases the knowledge on the unintentional negative impacts of agricultural practices on ecosystem service indicators, and then probably on their delivery, but also demonstrates that pesticide reduction is positively associated with proxies for ecosystem services. It also stresses the fact that these practices have to be implemented at both field and municipality levels.

## INTRODUCTION

Biodiversity threats are multiple, but agricultural landscapes are particularly affected, with farmland birds (Rigal et al., 2023), plants (Meyer et al., 2013; Storkey et al., 2011), earthworms (Blakemore, 2018), and bumblebees (Hemberger et al., 2021) sharply declining during the last decades. These impacts are partly due to pesticide use affecting non‐targeted organisms, thus decreasing invertebrate (Karimi et al., 2020; Leenhardt et al., 2023; Main et al., 2020; Schmidt‐Jeffris et al., 2021) and plant (De Snoo, 1999; De Snoo & Van der Poll, 1999; Schmitz et al., 2014) richness and abundance. Pesticides strongly structure plant and animal communities in agricultural landscapes by selecting the few species that are able to survive their use (Humann‐Guilleminot et al., 2025; Trager et al., 2013; Ziesche et al., 2024). Fertilization also reduces plant diversity in agroecosystems by favoring competitive fast‐growing species, thus homogenizing plant communities (Fried et al., 2009). However, habitat heterogeneity, including crop diversity, the presence of seminatural habitats, and agroecological infrastructures (hedges, flower or grass strips, field margins…) in these agricultural landscapes, can mitigate losses (Aviron et al., 2023; Boinot et al., 2023). These structures enhance connectivity by creating dispersal corridors. They also provide potential refuge zones, and diversify potential habitat and resources to sustain biodiversity (Priyadarshana et al., 2024; Sirami et al., 2019), including rare species (Fried et al., 2022). Seminatural habitats and landscape diversification therefore provide a key leverage to minimize agricultural impacts on biodiversity.

Field margins, for example, represent a shelter for biodiversity all year round, even when the crop is disturbed (Sorribas et al., 2016). They are defined as the uncultivated area that lies between a cultivated field and an adjacent landscape feature, such as a road, track, another habitat (e.g., grassland or forest), a field boundary (e.g., hedge or fence), or another cultivated field. These margins are not directly exposed to agricultural practices such as tillage, fertilization, or pesticide application within the plots. However, they can be affected indirectly by factors such as wind drift or runoff of chemical inputs. As a result, field margins form a distinct habitat separate from both the cultivated field and the adjacent habitat. They typically consist of a narrow strip of herbaceous vegetation dominated by perennial species and are often maintained through practices such as mowing. Field margins are key to delivering regulating ecosystem services in agricultural landscapes (Crowther et al., 2023; Mkenda et al., 2019). They host numerous plants that are absent or scarce in the field core, thus enriching plant communities present in agroecosystems and, for example, participating in maintaining threatened segetal species (Fried et al., 2024). The diversity of plant communities in field margins supports ecological processes such as pest regulation or crop pollination (Mkenda et al., 2019) by providing diverse food resources (nectar, seeds, preys…) that are not always present inside the cultivated crop fields (Aviron et al., 2023), such as floral resources in arable crop landscapes (Baude et al., 2016). Indeed, pollinators of agroecosystems depend strongly on wild plant species from adjacent habitats to forage (Balfour & Ratnieks, 2022), even when mass‐flowering crops are present (Crochard et al., 2022). Perennial plants have the potential to reduce soil erosion (Hanisch et al., 2020), which is a major agronomic problem (García‐Ruiz, 2010). Field margins composed of an important proportion of perennial species cover the soil all year long, specifically at critical periods for erosion, during fall and winter when most rainfall happens. Their presence prevents soil loss from the crop field, as it would be the case with only bare soil, therefore providing an additional ecosystem service of erosion reduction (Ali & Reineking, 2016). Field margins are therefore a crucial landscape element, and their management should be carefully optimized to enhance the delivery of key ecosystem services that support crop production.

Trade‐offs can appear between different ecosystem services, with the optimization of one service degrading the delivery of a different ecosystem service, or even increasing disservice delivery (e.g. favor floral resources vs reduce harvest as some pollinator‐friendly species, such as thistles, can also act as significant weeds) (Blanco et al., 2019; Yvoz et al., 2021). Farmers' fear of favoring pests or weeds when increasing plant biodiversity represents an important brake on the implementation of agroecological structures such as floral strips, hedges, or diversified field margins (Dumont et al., 2021). Therefore, assessing the role of field margins and plant biodiversity in ecosystem service delivery is necessary to shift agroecological practices in the right direction.

Field margin diversity is impacted by agricultural practices: recent studies demonstrated the large‐scale effects of pesticide use on field margin vegetation richness and composition at the French national scale (Fried et al., 2018; Henckel et al., 2025; Poinas et al., 2023). Pesticides could decrease vegetation‐based services' delivery from field margins by affecting their diversity (Winter et al., 2018). It is well known that management practices (e.g. mowing, tilling) and types of production (conventional vs organic) have impacts on biodiversity: intensification of these practices decreases both plant diversity and service delivery (Esposito et al., 2023; Fanfarillo et al., 2019; Winter et al., 2018). However, these effects are often studied at local or regional scales (Boinot & Alignier, 2023), by crop type (Mei et al., 2021), during 1 or 2 years (Boeraeve et al., 2020) and/or scarcely include multiple services and disservice evaluation. Studies specifically targeting field margins mostly focus on natural enemies or pests (Mkenda et al., 2019).

In this work, we aim at understanding the effects of agricultural practices, and more specifically of pesticide use, on indicators of vegetation‐based (dis)services (i.e., floral resources delivery, erosion control, plant conservation, and maintaining problematic weeds) through a nationwide‐scale monitoring of more than 450 French field margins, from 2013 to 2018. Field margin vegetation was surveyed annually around the main crop types (Andrade et al., 2021): cereals (with wheat and maize as the main crops), vineyards, and market gardening. We hypothesized that pesticide use at both field and municipality level would decrease services by reducing plant diversity. Additionally, fertilization would increase the presence of competitive and common ruderal agrotolerant plants. In conjunction with in‐field pesticide use, fertilization would positively affect disservices, as problematic weeds are mostly fast‐growing ruderal plants. Finally, a trade‐off between services and disservices could occur because factors increasing plant diversity could also increase the presence of problematic weeds.

## MATERIALS AND METHODS

**FIGURE 1:**
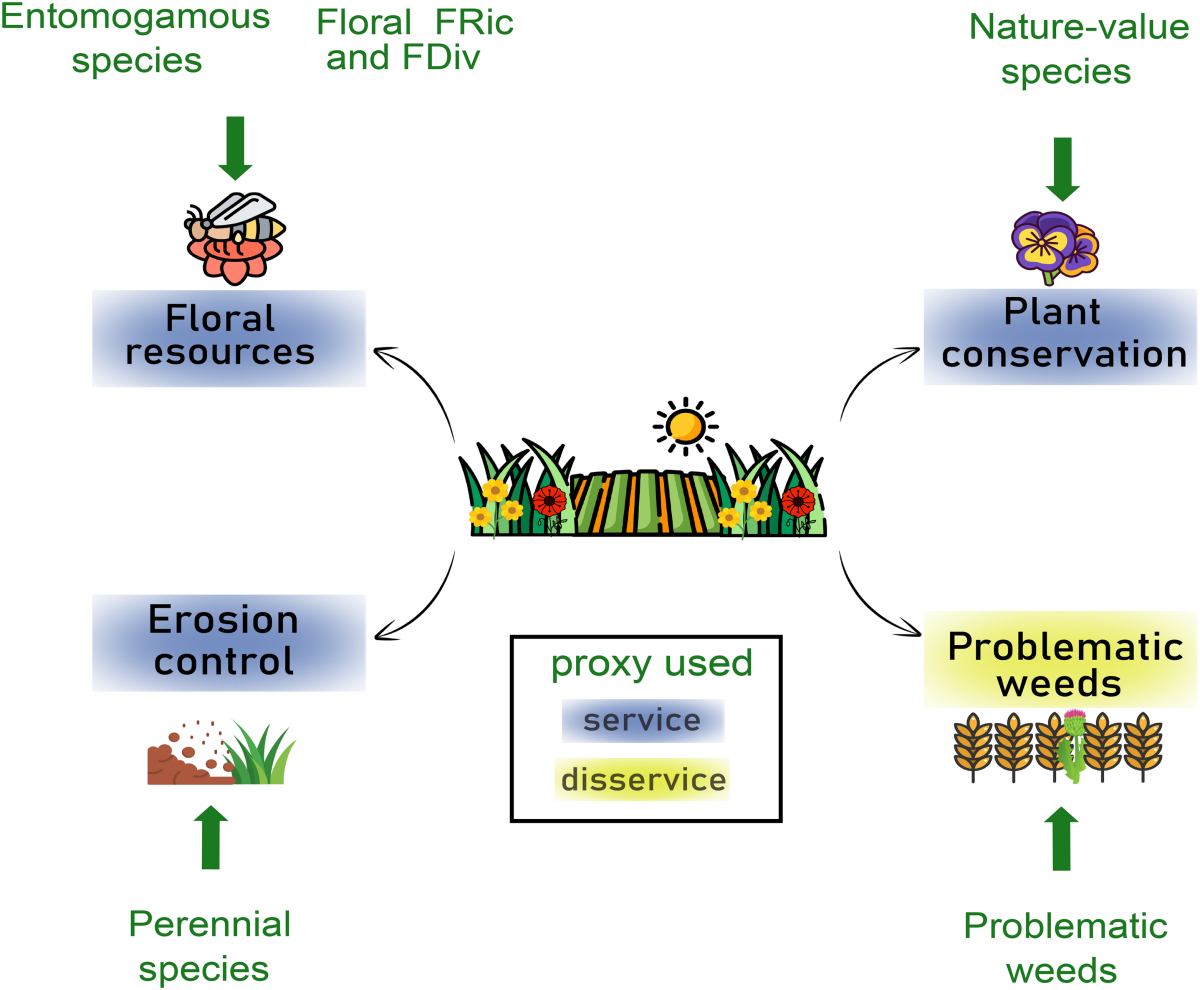
Proxies used to calculate the service and disservice indicators of field margins. FDiv, functional divergence; FRic, functional richness. This figure has been designed using resources from Flaticon.com (Icons by [Eucalyp], [Triberion], [Freepik], [Good ware], [Muhammad‐Usmad], [Manshagraphics], [Good ware], [Nsit]).

We quantified the potential delivery of multiple ecosystem (dis)services from plants in agricultural field margins through a standardized nationwide monitoring in France (Andrade et al., 2021), which is the European country with the largest agricultural production (Agreste, 2022). This study was conducted from 2013 to 2018 across 458 cereal, vineyard or market gardening field margins. Environmental and agronomic variables were also collected to understand the relationships between agricultural practices and (dis)service delivery. We focus on indicators for three potential ecosystem services and one disservice indicator (Figure 1): (1) the provision of floral resources estimated through the abundance of entomogamous plants and the floral functional richness (FRic) and divergence (FDiv) of field margin plants, (2) erosion control estimated through the abundance of perennial plants in field margin vegetation, (3) plant conservation, estimated through the abundance of “nature‐value” plants in field margin vegetation, and finally (4) the potential disservice of competition with the crop through the abundance of problematic weeds in field margin vegetation. Below, we describe the surveys and ecosystem service indicators used.

### Study sites and plant surveys

We used data from the 500ENI network, a standardized national biodiversity and agricultural practices monitoring program coordinated by the French Ministry of Agriculture that aims to describe the nontarget effects of agricultural practices on biodiversity (Andrade et al., 2021). For the purposes of this analysis, we used data gathered between 2013 and 2018, where plant communities in field margins were surveyed annually in 458 sites at their flowering peak, from May to July. The observers carrying out the botanical samplings are employees of either an agricultural chamber or plant health institutes (*FREDON*). They all have a background in either ecology, agronomy or both, and received botanical training on field margin vegetation. They were also given a botanical guide specifically compiled for the purpose of this 500ENI monitoring protocol, selecting the most common and abundant species, which are crucial to deliver ecosystem services. Each sampling was composed of 10 quadrats of 1 m^2^ (0.5 × 2 m) where species were recorded as present or absent (see Andrade et al., 2021 for more details on the samplings). For each sampling, each species therefore has a frequency score that varies between 1 and 10 which is used here as a proxy of abundance. Complementary analyses show that this frequency score is strongly and positively correlated with the abundance cover of each species (Appendix S1: Figure S1) and will be referred to as “plant abundance” in the rest of the analyses.

The study sites included three types of agroecosystems, which represent the main crops in France: 68% of sites were located in cereal margins (286), with wheat or maize as the main rotation crops (secondary crops included rapeseed, barley or sunflower in most sites), 22% in vineyards (101 sites), and 10% in market gardening fields (43 sites). Overall, 23% of the sites (108) were organic. Agricultural management in the fields adjacent to the margin surveyed was recorded annually through interviews with farmers for both in‐field and margin management practices. In‐field management information includes nitrogen fertilization dose (including both mineral and organic fertilization, as well as, for example, manures), copper dose, diversity of crop rotation (number of different crops over time divided by the number of years), and the treatment frequency index (TFI) for pesticide use. TFI is the cumulative ratio of the pesticide dose applied divided by the recommended dose (Halberg, 1999). Field margin practices include margin width, the number of management operations per year, and the nature of the operation (i.e., mowing with or without biomass collection). Each site was also characterized by its exposure (cf: shaded or not) and by an index of spillover risk defined by a function of topography and wind exposure as follows (Henckel et al., 2025):

(1) Spillover risk index=WE+S×SI,

where WE is the wind exposure (0, not exposed to wind; 1, wind‐exposed), S represents the position of the field margin in relation to the slope (0, not located at the bottom of a slope; 1, located at the bottom of slope), and SI is the slope index (0.5, 1%–10%; 1, 10%–20%; 1.5, 20%).

For the purposes of this study, field margins were defined as the uncultivated area of herbaceous vegetation between the cultivated field and the adjacent element (classified as built area or road, crop type, wood‐ hedgerow, grassland, grass strip, and wetland) opposite to the agricultural field. These were sampled once per year at the peak of the flowering season using 10 quadrats of 1 m^2^ located at the center of the field margin along a transect (the same every year). The presence or absence of each plant species and their occurrence over the 10 quadrats were recorded. Here, we used the total number of quadrats per transect as an index of relative abundance (from 0 = no occurrence in any quadrat along the transect; to 10 = occurs in every quadrat) (Poinas et al., 2023).

### Environmental characterization

To characterize the environmental context, we used climatic, soil, and landscape variables that have already been shown to be important drivers of plant communities of this network (Poinas et al., 2023). Climatic data were extracted from WorldClim (Fick & Hijmans, 2017) for each site and represent average climatic conditions over 30 years (1970–2000) at a 30‐sec resolution (roughly 1‐km resolution). The variables considered here were: mean annual temperature (in degrees Celsius), temperature annual range (degrees Celsius), and total rainfall of the year (in millimeters). Regarding soil variables, texture (in grams per square kilogram), pH, soil total nitrogen (in grams per square kilogram), and organic carbon (in grams per square kilogram) content were directly measured in the field for 427 sites; for the remaining sites, the same variables were extracted from SoilGrids (https://soilgrids.org/) at 250‐m resolution and a depth of 0–5 cm (Hengl et al., 2014). Soil bulk density (grams per cubic centimeter) was extracted from SoilGrids for all sites.

Landscape composition was considered at a 1‐km buffer around the field margins. Spatial data were treated using QGIS v. 3.14.16 and R v. 4.0.1 with the package ALM (Allart et al., 2021). Spatial data sources consisted of freely accessible databases: the French Land Parcel Identification System (RPG) from the common agricultural policy (CAP) (see: https://www.data.gouv.fr/fr/datasets/registre‐parcellaire‐graphique‐rpg‐contours‐des‐parcelles‐et‐ilots‐culturaux‐et‐leur‐groupe‐de‐cultures‐majoritaire), the BD TOPO database of the French National Institute of Geographic and Forest Information (IGN) (see: https://geoservices.ign.fr/documentation/donnees/vecteur/bdtopo), and the OSO Land Cover from Theia (see: https://artificialisation.developpement-durable.gouv.fr/bases-donnees/oso-theia). The proportion of each of the following landscape components was quantified within the 1‐km radius surrounding the field margins: crops, wet area, buildings and roads, and seminatural habitats, calculated as the sum of the percentage of forests, grasslands, hedges, field margins and natural mineral areas.

Finally, we used three variables describing agricultural practices at the municipality level (i.e., the “commune,” which is the smallest administrative level in France): the percentage of organic fields of the total agricultural surface, mean herbicide TFI, and mean total TFI (sum of herbicide, fungicide, and insecticide TFIs) (Solagro, 2022, see: https://solagro.org/nos-domaines-d-intervention/agroecologie/carte-pesticides-adonis).

### Trait data and indicators of (dis)services

Using the botanical samplings we measured six indicators of (dis)services, calculated on the abundance of the species present in at least 1% of the field margin surveys (Figure 1). These indicators were used to assess floral resource, functional floral diversity, erosion control, conservation interest, and a disservice related to the presence of problematic weed species. For floral resources we used as an indicator the proportion of strict and optional entomogamous species (i.e., each plant was classified as either strictly entomogamous, strictly anemogamous, or optional entomogamous), in each community. The mode of pollination was extracted from Baseflor (Julve, 1998). To calculate floral FRic and FDiv, we considered flower color (i.e., *white, yellow, pink, green, blue, purple*), number of colors per flower, flowering onset, flowering duration, and inflorescence type, all extracted from Baseflor (Julve, 1998), presence of nectar from BiolFlor (Kühn et al., 2004), and floral symmetry (Botineau, 2010), all traits known to be linked to pollinator presence (Michelot‐Antalik et al., 2025). These six traits were used as proxies of floral functional diversity according to Villéger et al. (2008). We used the proportion of perennial plant species (Julve, 1998) as an indicator for erosion control since perennial species tend to stabilize the soil (Hanisch et al., 2020). For conservation interest, we recorded the proportion of nature‐valued species in each community, as defined by Aavik and Liira (2009), that is, the species present in less than 10% of agricultural field cores (for which field margins are essential for population persistence), evaluated in France with a separate dataset (Fried et al., 2018). Finally, to assess the disservice of weed reservoir, we recorded the proportion of problematic weeds in each field margin community. Each species was considered either problematic or not for annual crops, vineyards and market gardening according to previous expert assessments (ACTA, 2014; Maillet et al., 2001). The proportion of each group in each survey was calculated based on the abundance scores of each species (species abundance in the survey divided by the total abundance of all species confounded). As an example, if we only have two species in the sampling (i.e., in the 10 quadrats sampled in the margin), one identified as a problematic weed with an abundance of 4, and one not identified as a problematic weed, with an abundance of 6, then the proportion of problematic weeds would be 40%.

### Statistical analyses

**FIGURE 2:**
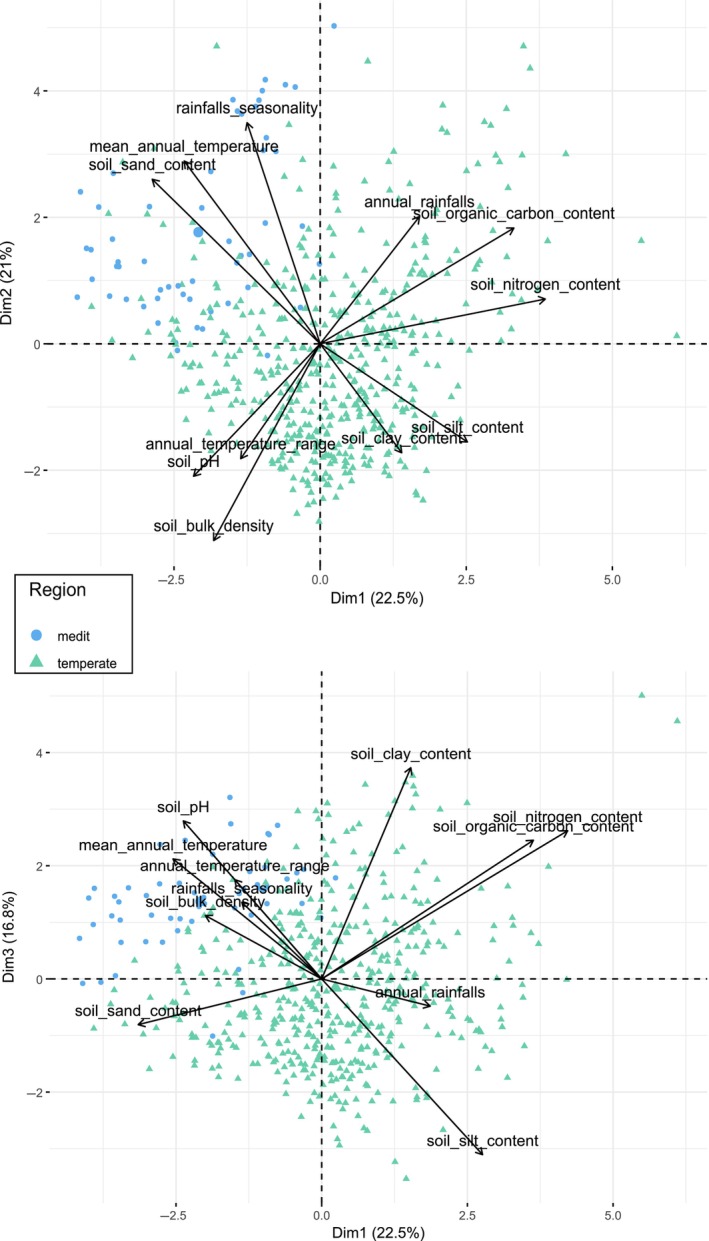
Axes of soil and climatic principal components analysis (PCA) ran on soil and climatic variables from the 458 studied fields in this study, whose coordinates on the three axes were extracted in order to use them as soil and climatic variables added in the models. (A) First and second axes. (B) First and third axes.

We used generalized linear mixed models with each one of the six ES (ecosystem services) indicators (i.e., proportion of entomogamous species as quantity of floral resources; floral FRic and floral FDiv as diversity of floral traits; proportion of perennial species as erosion reduction; proportion of nature‐value species as plant diversity conservation; and proportion of problematic weeds as disservice of competition with the crop) separately as response variables. Sites were incorporated as random effects, since each site was surveyed every year during the study period. All the predictors were scaled before analysis to allow comparison between coefficient estimates. We used a hierarchical approach, incorporating different sets of variables progressively in the models, because at this large scale of analysis the climatic and landscape variables explain more plant variability than local variables, such as agricultural practices (Poinas et al., 2023). As our main goal was to understand the impacts of in‐field agricultural practices, we used this step‐by‐step model approach to avoid overestimation of management effects when other macroecological variables are at play. We started with a model (“m0”) that included only the general predictors describing the sites to consider all confounding effects: crop type, spillover risk, exposure, year, and Julian day of the date corresponding to the plant survey. We then used a multiple‐step model selection framework (function “dredge”) to add the soil, climatic, landscape and agricultural management predictors to the initial model, starting from large scale (municipality‐level variables) to local (on‐site variables). We first added the soil and climatic variables represented by the first three axes of the principal components analysis (PCA) (Figure 2) (“m1” models); then the landscape variables: percentage of crops, building roads, wet areas and seminatural habitats in a 1‐km radius, and the percentage of organic field on total municipality field surface (“m2” model); then we added pesticide use at the municipality level: mean herbicide TFI and mean total TFI (“m3” models); then we added field margin management: margin width, number of management operations per year, and the nature of the operation (i.e., mowing with or without biomass collection) (“m4” models); and finally the agricultural practices applied in the fields adjacent to the surveyed margins: herbicide TFI, fungicide‐insecticide TFI (sum of fungicide and insecticide TFIs), nitrogen fertilization dose, copper dose, and diversity of crop rotation (“m5” models). To test the impact of production type (organic vs. conventional) and because this variable was correlated with all the agricultural practices, we also ran an alternative model with production type only instead of agricultural practices. Correlation of fixed effects was verified with the variance inflation factor (VIF) before running the models. Predictors with VIF values of 5 or higher, indicating multicollinearity issues, were removed. At each step, we performed model stepwise comparisons and kept the model with the lowest Akaike information criterion value (AIC_c_) corrected for small sample sizes (Burnham & Anderson, 2004). The threshold to consider two models as different was ΔAIC_c_ > 2 (Burnham & Anderson, 2004). In case of equal AIC_c_, we selected the most parsimonious, with the lowest number of fixed effects. We used likelihood ratio tests to assess the selected models and calculated the marginal and conditional *R* ^2^ (Nakagawa & Schielzeth, 2013).

All statistical analyses were run using R version 4.3.2 (R Core Team, 2023), and in particular the packages *lme4* (Bates et al., 2015), *vegan* (Oksanen et al., 2007), *FD* (Villéger et al., 2008), *FactoMineR* (Lê et al., 2008), *MuMIn* (Barton, 2009), *car* (Fox et al., 2012), and *terra* (Hijmans et al., 2022). All scripts and data to replicate the analysis can be found in Genty (2024).

## RESULTS

### Soil and climatic PCA

We decided to retain the first three axes of the PCA as soil and climatic explanatory variables, which account for slightly more than 60% of the total variance. The first PCA axis (22.5% of variance) was positively related to soil nitrogen and organic carbon content, percent of silt in the soil, and temperate region, while it was negatively linked to percent of sand in the soil, mean temperatures, and the Mediterranean region. This axis clearly differentiates between Mediterranean and temperate zones. The second PCA axis (21% of variance) was positively related to rainfall seasonality, mean temperature, percent of sand in the soil, and partly to the Mediterranean region, while being negatively correlated to soil bulk density and the temperate region. This axis partitions the sites between those located in littoral vs continental regions. The third PCA axis (16.8% of variance) was positively linked to percent of soil clay, pH, nitrogen, and organic carbon content and to both the Mediterranean and temperate regions and is negatively correlated to percent of silt in the soil and to temperate regions. This axis differentiates between sites located in more fertile soils from those in poorer ones.

### Effects of environmental and agronomic predictors on (dis)services indicators

The proportion of perennial and nature‐value species was positively correlated (Pearson's *R* = 0.54), while those of nature‐value species and those of problematic weeds were negatively correlated (Pearson's *R* = −0.41). None of the other indicators were correlated above *R* = 0.25.

**FIGURE 3:**
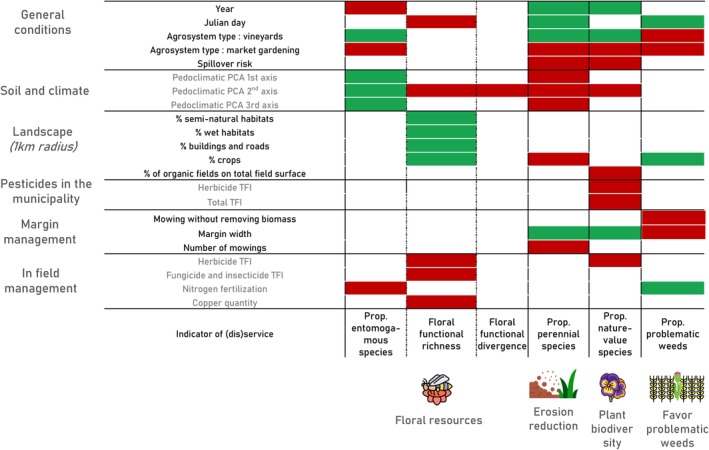
Results of the generalized mixed models: Effects of the predictors on the (dis)services of the field margin plant communities. The field was always added in the model as random variable. The positive effects are drawn in green and the negative effects are drawn in red. FDiv, functional divergence; FRic, functional richness, TFI, treatment frequency index. This figure has been designed using resources from Flaticon.com.

We present the results below starting from the broader scale predictors and going to the most local predictors. All the detailed results of the models can be found in Figure 3 and Table 1. In what follows, we only mention the relationships that came out as significant, and omit the rest.

**TABLE 1:** Individual effects of significant predictors of field conditions, soil and climatic variables, pesticide use at the municipality scale, field margin management and in‐field agricultural practices on the indicators of (dis)services.

Ecosystem service indicator	Predictor	Estimate	SE	*p* value	Signif	*R* ^*2*^ *m*	*R* ^*2*^ *c*
Proportion of entomogamous species	Year	−0.005	0.002	0.059	Trend	0.22	0.57
	Crop type : market gardening	−0.012	0.02	<0.001	***		
	Crop type : vineyard	0.084	0.018	<0.001	***		
	Soil and climatic PCA 1st axis	0.012	0.004	0.004	**		
	Soil and climatic PCA 2nd axis	0.015	0.004	<0.001	***		
	Soil and climatic PCA 3rd axis	0.016	0.005	0.001	***		
	**N fertilization dose**	**−0.038**	**0.007**	**<0.001**	***		
Functional floral richness	Julian day	−0.005	0.001	<0.001	***	0.06	0.47
	Soil and climatic PCA 2nd axis	−0.002	0.001	0.03	*		
	% semi−natural habitats in the landscape	0.103	0.034	0.002	**		
	% wet habitats in the landscape	0.023	0.008	0.003	**		
	% roads and buildings in the landscape	0.056	0.018	0.002	**		
	% crops in the landscape	0.116	0.038	0.002	**		
	**In field fungicide and insecticide TFI**	**−0.004**	**0.001**	**0.003**	**		
	**In field herbicide TFI**	**−0.003**	**0.001**	**0.028**	*		
	**Copper use**	**−0.002**	**0.001**	**0.004**	**		
Functional floral divergence	Soil and climatic PCA 2nd axis	−0.005	0.001	<0.001	***	0.01	0.21
Proportion of perennial species	Year	0.725	0.27	0.007	**	0.18	0.54
	Julian day	3.77	0.459	<0.001	***		
	Spillover risk	−1.726	0.585	0.003	**		
	Crop type: market gardening	−5.343	2.293	0.014	*		
	Soil and climatic PCA 1st axis	−2.888	0.484	<0.001	***		
	Soil and climatic PCA 2nd axis	−1.284	0.45	0.004	**		
	Soil and climatic PCA 3rd axis	−0.981	0.502	0.051	Trend		
	% crops in the landscape	−2.444	0.681	<0.001	***		
	Margin width	2.252	0.611	<0.001	***		
	Number of mowings	−0.705	0.35	0.044	*		
Proportion of nature−value species	Year	0.963	0.254	<0.001	***	0.13	0.49
	Spillover risk	−2.45	0.535	<0.001	***		
	Crop type : market gardening	−7.751	2.014	<0.001	***		
	Soil and climatic PCA 2nd axis	−1.137	0.405	0.005	**		
	% of organic fields on total field surface	−1.865	0.659	0.005	**		
	**Municipality herbicide TFI**	**−3.009**	**0.79**	**<0.001**	***		
	**Municipality total TFI**	**−2.569**	**0.727**	**<0.001**	***		
	Margin width	2.114	0.554	<0.001	***		
	**In field herbicide TFI**	**−1.648**	**0.636**	**0.01**	**		
Proportion of problematic weeds	Julian day	1.43	0.403	<0.001	***	0.16	0.49
	Crop type: market gardening	−3.842	1.951	<0.001	***		
	Crop type: vineyard	−10.53	1.639	<0.001	***		
	% crops in the landscape	1.762	0.576	0.002	**		
	Mowing without removing biomass	−1.827	0.571	0.001	**		
	Margin width	−1.121	0.537	0.037	*		
	**N fertilization dose**	**1.457**	**0.656**	**0.027**	*		

*Note*: Effects linked to agricultural practices are in bold. Field is defined as random effect for all models. Marginal *R* ^2^ (*R* ^2^ *m*) represents the proportion of variance explained by fixed effects in the model. Conditional *R* ^2^ (*R* ^2^ *c*) includes random effects. *p*‐values and significance stars are from the type II ANOVA's Chi^2^. *p < 0.05, **p < 0.05 and > 0.001, ***p < 0.001.

Abbreviations: N fertilization: nitrogen fertilization; PCA, principal components analysis; TFI, treatment frequency index.

General conditions all affected at least one service. The proportion of perennial and nature‐value species increased over time (variable “year”) while those of entomogamous species decreased. Margins sampled later in the year had lower floral FRic but a higher proportion of perennial species and problematic weeds. Spillover risk drove margins with a lower proportion of perennial and nature‐value species. Market gardening margins were composed of fewer entomogamous, perennial, and nature‐value species than other crops; vineyard margins were composed of more entomogamous species, and cereal margins were composed of more problematic weeds.

The three axes of the soil and climatic PCA affected positively the proportion of entomogamous species but negatively those of perennial species. Axis 2 impacted negatively all the other service indicators but did not affect the proportion of problematic weeds.

Landscape variables impacted three (dis)service indicators. All landscape components (percentage of seminatural habitats, of wet areas, of crops and of buildings and roads) positively affected floral FRic. The percentage of crops in the landscape negatively affected the proportion of perennial species. Finally, the percentage of organic fields at the municipality scale negatively impacted the proportion of nature‐value species.

Pesticide use at the municipality scale did not affect any (dis)service indicator except the proportion of nature value, which decreased with both total and herbicide TFIs at the municipal scale.

Margin management affected the proportion of perennial, nature‐value, and problematic weed species. Margin width had a positive effect on communities composed of more perennial and nature‐value species and of fewer problematic weeds. There was a lower proportion of perennial species in more regularly mown areas and fewer problematic weeds in margins where the farmers did not remove the plant biomass after mowing.

Finally, the in‐field agricultural practices affected all indicators except floral FDiv and the proportion of perennial species. Nitrogen fertilization dose negatively affected the proportion of entomogamous species while it was positively linked to those of problematic weeds. Floral FRic was reduced by copper quantity, as well as by in‐field herbicide and non‐herbicide TFIs. Finally, in‐field herbicide TFI also reduced the proportion of nature‐value species. Alternatively, in the model where production type was tested instead of the in‐field agricultural practices, organic production was positively associated with the proportion of entomogamous species, floral Fric, and the proportion of nature‐value species (Appendix S1: Table S1).

The conditional *R* ^2^ was 0.22 for the proportion of entomogamous species, 0.06 for floral FRic, 0.01 for floral FDiv, 0.18 for the proportion of perennial species, 0.13 for those of nature‐value species, and 0.16 for those of problematic weeds.

## DISCUSSION

Although other studies have shown negative impacts of intensive agricultural practices on vegetation‐based services in agricultural landscapes (Esposito et al., 2023; Winter et al., 2018; Wittwer et al., 2021), this is one of the first studies to do so at a national scale. Overall, our results are consistent with our expectations that intensive agricultural practices, mainly pesticide use and fertilization, would decrease field margin's ecosystem service indicators. These impacts could be driven by local in‐field or municipality‐level agricultural practices. Fertilization negatively affected floral resource delivery, which was also the ecosystem service indicator that seemed to be the most affected by agricultural intensification. Contrary to our expectations, we did not find trade‐offs between service proxies and the indicator of disservice that we studied.

### Pesticide use affects the potential of field margins to deliver services

Globally, 74% of world agricultural areas are already contaminated with at least one pesticide, and up to 91% in Europe (Tang et al., 2021). Several studies have suggested that plant diversity and ecosystem service provision are higher when pesticide use is reduced or null (Nascimbene et al., 2012; Ostandie et al., 2021; Wittwer et al., 2021). In our study, we clearly demonstrated negative effects of pesticide use on ecosystem service indicators, all over France and for three studied crops (i.e. *cereals, vineyards, market gardening*). We notably found more negative effects of herbicide use, indicating that herbicides are the most damaging type of pesticide for vegetation‐based services as evaluated in this work. However, fungicides and insecticides also affected floral FRic and the presence of nature‐value plants. Few studies have already explored the effects of fungicides on plants or on terrestrial arthropod biodiversity (Giffard et al., 2022), and these potential impacts need to be evaluated further. While herbicides are used to reduce weed abundance and minimize competition with crops, our results showed that their use can also decrease the diversity of field margin plant communities by diminishing the presence of so‐called “nature‐value” species, that are most of the time less ruderal and more grassland adapted, probably less prone to cause damage to crops. This unintended effect of herbicide use at both scales can result in a homogenization of plant communities and in the dominance of few species, driving a more competitive weed community (Esposito et al., 2023). This result is in line with many studies showing that herbicide use drives a decline in local plant biodiversity at different scales and in different countries (Carmona et al., 2020; Gaba et al., 2016; Tarifa et al., 2021). It is important to notice that the service proxies were also positively impacted by organic farming, showing that, overall, pesticide use reduction is associated with functionally richer plant communities, with more entomogamous and nature‐value species, that are complementary to weeds and increase plant diversity at the landscape level.

Our results are in line with a previous study based on the same 500 ENI network, but with only 2 years of data (2013 and 2014), where fertilization, along with herbicide TFI, was also a major driver of plant functional composition in general (Fried et al., 2018). Fertilization is regularly found to negatively affect spontaneous vegetation in agroecosystems (Kleijn & Verbeek, 2000; Meyer et al., 2013), as in our current study.

### Trade‐off between the studied services and disservice

Another central question of this work was the possible trade‐offs between services, or between services and disservices. We did not find any trade‐off regarding the impacts of agricultural practices having opposite effects on ecosystem service indicators, favoring one while diminishing the other. In our work, the same agricultural practices seemed to either decrease disservices and increase services or to only affect one of the indicators and not the others. For example, larger margin width favors plant diversity and erosion reduction while reducing the presence of problematic weeds. Also, fertilization favors problematic weeds while disfavoring floral resources. Therefore, for the (dis)services studied here, the same management recommendations can be made to both increase service delivery and decrease disservice. This is contrary to what was found by Yvoz et al. (2021) for weed communities within the field, where one community could not deliver resources for auxiliaries and minimize competition with the cultivated plant at the same time.

Another interesting finding in our work is that ecosystem services were not affected by practices at the same scale (Figure 3). Only the nature‐value species were impacted by the agricultural practices at the municipal scale: agricultural intensification at the municipal scale seemed to impoverish the species pool and to promote more homogenized and agrotolerant plant communities. However, the in‐field herbicide use also decreased the presence of nature‐value species, showing that pesticide reduction has to be implemented at both scales (on site and regional) to preserve plant richness and maintain ecosystem services. On the other hand, floral resources seemed more driven by both soil and climatic conditions, landscapes, and in‐field agricultural practices, and less by intensification at a higher spatial scale than the field.

Overall, in French field margins, plant communities providing floral resources and hosting less common species are also composed of less competitive weeds. This is in line with the work of Adeux et al. (2019) who demonstrated that weed communities with higher evenness cause less damage, even when they present similar species richness. This is also in line with the concept of “neutral weed communities” (Esposito et al., 2023), that is, the idea that promoting satisfactory yield, spontaneous plant biodiversity and ecosystem services is possible at the same time. However, all the cited studies were following weed communities within the agricultural fields, whereas we focus on the margins, where management practices can be applied somewhat independently from optimization of the adjacent crop yield and plants are not in direct competition with crops.

### Providing floral resources for insects: A particularly threatened ecosystem service

The presence of floral resources was a particularly affected service indicator because the proportion of entomogamous species was reduced by fertilization quantity. Previous studies have already found that ruderal and agrotolerant species, favored by intensification, are preferentially autogamous (Fried et al., 2024) and that weed communities are less entomogamous when they are more disturbed (Tarifa et al., 2021). Floral resources' quality was also threatened by pesticide use since floral FRic, which is crucial to deliver flowers to pollinators (Fornoff et al., 2017), decreased with higher pesticide use. Agricultural intensification was found in our study to both decrease the presence of floral resources and to functionally impoverish it. However, functional floral divergence was not explained by any predictor tested here. Agricultural practices such as mowing or fertilization have already been found to impact floral resources (Goulnik et al., 2020; Rotchés‐Ribalta et al., 2023) but very few studies had already looked at the relationships between pesticide use and floral traits. We demonstrated here that FRic of floral resources is not only affected by agricultural practices such as mowing or grazing, as already shown in grasslands, but also by fertilization and pesticide use.

Another impact of agricultural intensification on floral resources, and then on flower‐visiting insects, that is not measured in this work, is the risk of contaminating the whole trophic chain through the consumption of floral resources that are contaminated with pesticides (Tison et al., 2024; Ward et al., 2022). It therefore seems crucial to implement solutions to decrease their impacts on surrounding biodiversity. Some have proposed, for example, the implementation of untreated crop edges to preserve or increase floral resources, which are essential for plant pollination in agricultural landscapes (Crochard et al., 2022). These types of solutions need to be considered and studied further to minimize agricultural impacts on field margin vegetation.

## CONCLUSION

The positive effect of organic production on ecosystem services was already known for weed communities (Boeraeve et al., 2020; Rotchés‐Ribalta et al., 2023; Tuck et al., 2014; Winter et al., 2018). In this work, we demonstrated for the first time at a national scale and in three crop types, that pesticide use and/or fertilization dose have consistent negative effects on ecosystem (dis)services delivered by field margin vegetation, including on species that are not considered target and that can represent an increase in agricultural biodiversity at the landscape scale. This finding is crucial to guide stakeholders toward the implementation of agricultural practices that are less harmful and to deliver crucial ecosystem services by favoring wild plant diversity through increasing margin width and by keeping an important distance between the sprayed field core and the margin.

## CONFLICT OF INTEREST STATEMENT

The authors declare no conflicts of interest.

## Supporting information


Appendix S1.

## Data Availability

Data and code (Genty 2024) are available in Zenodo at https://doi.org/10.5281/zenodo.13737015.
