# Supplementary material for: Intensive management negatively impacts field margin ecosystem service indicators at both field and landscape levels
Source: Ecol Appl. 2026 Jan 6;36(1):e70161. doi: 10.1002/eap.70161 (PMC12823288; doi:10.1002/eap.70161)
Supplement: Supplementary file 1 — Appendix S1. [file EAP-36-e70161-s001.pdf]

Appendix S1

Intensive management negatively impacts field margin ecosystem service indicators at both field and landscape levels

Léa Genty, Christine N. Meynard, Marie-Charlotte Bopp, Laura Henckel, Aurélien Chayre, Caroline Gibert, Guillaume Fried

in *Ecological Applications*

a.

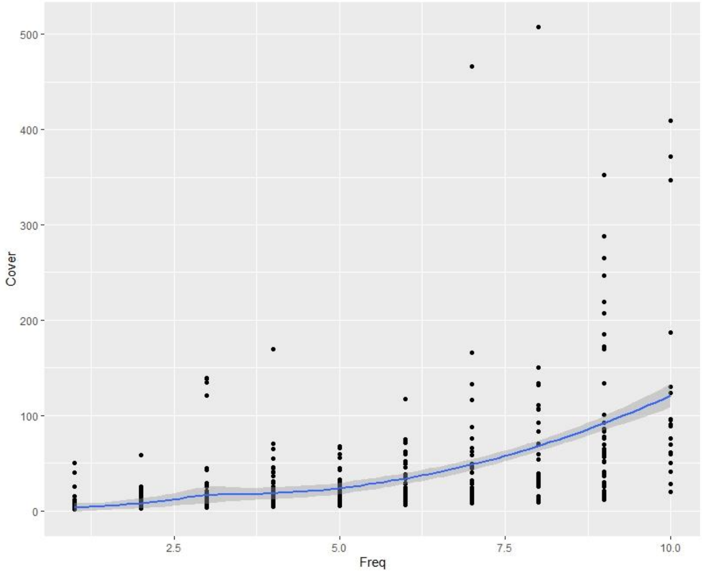

b.

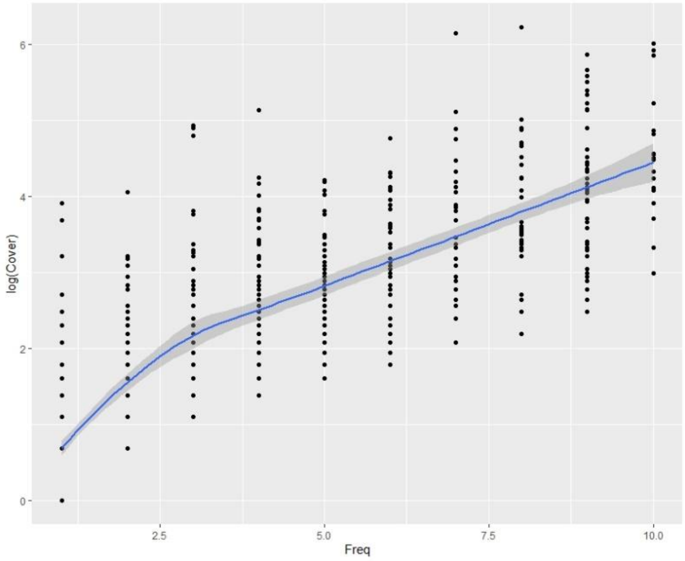

Figure S1. Relationship between (a) plant cover and plant frequency (Pearson's R: 0.6, Spearman's R: 0.8) and (b) log of plant cover and plant frequency, (Pearson's and Spearman's R: 0.8) as measured for the 500ENI network. Plant cover is the sum of abundance of each species in the 10 quadrats. This figure is based on a sampling of 8 field margins at three different dates according to both methods: plant cover as the % of abundance and plant frequency, used as proxy of abundance in the manuscript.

Table S1. Individual effects of significant predictors of field conditions, pedoclimatic variables, pesticide use at the municipality scale, field margin management and production type (organic vs conventional) on the indicators of (dis)services for which production type was significant. Effects linked to production type are in bold. Field is defined as random effect for all models. Marginal R2 (R2m) represents the proportion of variance explained by fixed effects in the model. Conditional R2 (R2c) includes random effects. P-values and significance stars are from the type II ANOVA's Chi2.

Ecosytem service indicator	Predictor	Estimate	Std error	p value	signif	R2m	R2c
Proportion of entomogamous species	Year	-0.005	0.002	0.059	trend	0.2	0.57
	Crop type : vineyard	0.13	0.016	<0.001	***		
	Pedoclimatic PCA 1st axis	0.013	0.016	<0.001	***		
	Pedoclimatic PCA 2nd axis	0.013	0.004	0.002	**		
	Pedoclimatic PCA 3rd axis	0.018	0.005	0.001	***		
	**Conventional production**	**-0.046**	**0.013**	**<0.001**	*****		
Functional floral richness	Julian day	-0.005	0.001	<0.001	***	0.05	0.47
	Pedoclimatic PCA 2nd axis	-0.003	0.001	0.011	*		
	% semi-natural habitats in the landscape	0.116	0.034	<0.001	***		
	% wet habitats in the landscape	0.026	0.008	<0.001	***		
	% roads and buildings in the landscape	0.061	0.018	<0.001	***		
	% crops in the landscape	0.130	0.038	<0.001	***		
	**Conventional production**	**-0.009**	**0.003**	**0.007**	****		
Proportion of nature-value species	Year	0.957	0.254	<0.001	***	0.13	0.49
	Spillover risk	-2.45	0.535	<0.001	***		
	Crop type : market gardening	-6.664	1.969	<0.001	***		
	Pedoclimatic PCA 2nd axis	-1.211	0.404	0.003	**		
	% of organic fields on total field surface	-2.22	0.689	0.001	**		
	Municipality herbicide TFI	-3.328	0.785	<0.001	***		
	Municipality total TFI	62.695	0.727	<0.001	***		
	Margin width	2.163	0.554	<0.001	***		
	**Conventional production**	**-3.263**	**1.393**	**0.02**	***		
